# Impact of clinical and dosimetric factors on severe oral mucositis in head and neck cancer: insights from a phase II clinical trial

**DOI:** 10.3389/fonc.2025.1679589

**Published:** 2025-11-17

**Authors:** Alicia Lozano-Borbalas, Olivia Jordi-Ollero, Jordi Marruecos, Nuria Farre, Isabel Planas, Maria Dolores Toledo, Ricard Mesia, Arturo Navarro-Martin

**Affiliations:** 1 Radiation Oncology Department, Catalan Institute of Oncology, L´Hospitalet de Llobregat, Spain; 2 Universitat de Barcelona, Barcelona, Spain; 3 Institut d’Investigacio Biomedica de Bellvitge, Barcelona, Spain; 4 Physics Department, Catalan Institut of Oncology, L´Hospitalet de Llobregat, Spain; 5 Radiation Oncology, Catalan Institute of Oncology, Girona, Spain; 6 Radiation Oncology, Hospital de la Santa Creu i Sant Pau, Barcelona, Spain; 7 Radiation Oncology Department, Catalan Institute of Oncology, Badalona, Spain; 8 Radiation Oncology Department, Hospital Universitario Virgen de la Victoria, Málaga, Spain; 9 Instituto de Investigacion Biomedica de Malaga, Málaga, Spain; 10 Plataforma en Nanomedicina-Instituto de Investigacion Biomédica de Malaga y plataforma en nanomedicina (IBIMA) Plataforma Bionad, Málaga, Spain; 11 Medical Oncology Department, Catalan Institute of Oncology, Badalona, Spain; 12 B.ARGO Badalona Applied Research Group in Oncology, Badalona, Spain

**Keywords:** oral mucositis, radiotherapy, cetuximab, cisplatin, pharyngeal mucositis

## Abstract

**Introduction:**

Oral mucositis (OM) is the most common acute treatment-limiting adverse effect in patients with head and neck cancer (HNC), particularly following concomitant radiotherapy (RT) and systemic therapy. However, the effects of clinical and dosimetric parameters on the onset of severe OM remain controversial. We aimed to determine the association between clinical and dosimetric parameters and severe OM in the oral and pharyngeal mucosae in a randomized phase II clinical trial.

**Patients and methods:**

A subgroup analysis of data from a clinical trial was conducted to assess the efficacy of a 3% melatonin oral gel (Mucomel^R^) to prevent OM in patients with HNC. A total of 54 patients treated with intensity-modulated radiotherapy (IMRT) (66–69.96 Gy/33 fractions) plus concomitant systemic therapy (cisplatin or cetuximab) +/− melatonin rinses were included. The association between clinical and dosimetric parameters and grade (G) ≥3 OM was determined. For this analysis, the oral mucosa was divided into the oral and pharyngeal mucosae.

**Results:**

The following variables were significantly associated with G3 OM in the oral mucosa: oropharyngeal localization (*p* = 0.03), treatment with cetuximab (*p* = 0.01), oral mucosa volume included in low planning target volume (PTV) (PTV1: 54.12 Gy) and intermediate treatment doses (PTV2: 60 Gy), V35 >70% (*p* = 0.007), and a median RT dose of 56.6 Gy (*p* = 0.02). The absolute healthy volume of the oral mucosa was a significant protective factor (*p* = 0.03; McFadden’s pseudo-*R*^2^ = 0.46). None of the clinical or dosimetric variables was significantly associated with G3 OM in the pharyngeal mucosa.

**Conclusion:**

Oropharyngeal cancer, cetuximab, and low and intermediate RT dose to the oral cavity mucosa were significantly associated with the onset of severe oral mucositis. Given the association between these previous factors with a higher risk of G3 OM, they should be considered during treatment planning and dosimetry in patients treated with cetuximab for oropharyngeal cancer.

## Introduction

Oral mucositis is the most common acute complication in patients with head and neck cancer (HNC) undergoing radiotherapy (RT) in combination with systemic therapy. Over 90% of these patients develop at least grade 2 (G2) oral mucositis (OM) and up to 56% experience severe (G3–4) OM (1–3).

The pathophysiology of OM is highly complex and multifactorial. Several biomarkers, including epidermal growth factor (EGF), C-reactive protein, TNF-alpha, and cytokines, have been linked to an increased incidence and severity of mucositis (4–6). Nevertheless, despite their identification, none of them has been translated into clinical practice.

Despite multiple proposed strategies (drugs, natural products, antioxidants, antiseptics, etc.) (7–11) to prevent or manage OM, clinical trials conducted to date have failed to demonstrate significant efficacy in reducing treatment-related OM (12–14).

The most effective method remains reducing radiation exposure to healthy mucosae, particularly through advanced techniques such as IMRT. Numerous retrospective studies have investigated the relationship between the development of OM and RT dose and treatment volume (15–18), leading to clinical guidelines that propose recommended dose constraints (19, 20). The main challenge in dose reduction lies in maintaining a therapeutic dose to the target without compromising treatment efficacy, which has driven interest in dosimetric strategies to reduce doses in healthy tissues.

An important advantage of IMRT over less advanced RT techniques is that it permits the individual evaluation of dosimetric parameters to the oral and pharyngeal mucosae as a function of the target location. In this regard, it would be valuable to assess the influence of dosimetric factors on the onset of OM in each region separately, which would allow us to perform more comprehensive dosimetric studies to better determine how to best prevent mucositis.

In this context, the objective of this exploratory study was to evaluate the association between grade ≥3 oral mucositis (OM) and dosimetric parameters, specifically radiation dose and volume, in the oral and pharyngeal mucosa, as assessed by the Radiation Therapy Oncology Group (RTOG) (21) and Common Terminology Criteria for Adverse Events 4th version (CTCAE v4.0) (22) criteria. Another objective was to explore the relationship between clinical variables and the development of OM in this patient population.

For this purpose, we analyzed treatment planning data from a previously conducted phase II clinical trial (NCT02630004) (23) led by our group, which investigated the efficacy of Mucomel^R^ in preventing OM in patients with head and neck cancer receiving systemic therapy (cisplatin or anti-EGFR agents) combined with radiotherapy. Although the trial did not meet its primary endpoint, Mucomel^R^ failed to significantly reduce the incidence of grade 3–4 OM according to the RTOG scale (53% vs. 64%, *p* = 0.36); however, it provided valuable clinical and dosimetric data that form the basis of the current analysis.

## Materials and methods

This study was based on data from a subset of patients included in our previous phase II trial (23). In the present exploratory study, we included only the patients for whom dosimetric data were available (*n* = 54). Demographic and clinical characteristics of the final sample are shown in **Table 1**.

**Table 1:** Clinical and demographic characteristics of the sample (*n* = 54).

Variable	*N* (%)*
Median age, years (range)	59.7 (84–44)
Sex, male	49 (91)
Systemic treatment	
Cetuximab	27 (50)
Cisplatin	27 (50)
Tumor location	
Oral cavity	13 (24)
Oropharynx	28 (52)
Larynx	4 (7.4)
Hypopharynx	5 (9.0)
Nasopharynx	4 (7.4)
Stage	
III	5 (9.2)
IVA	41 (76)
IVB (except the nasopharynx)	8 (15)
Treatment	
Systemic therapy + RT	42 (78)
Surgery + systemic therapy + RT	12 (22)
Mucomel	
Yes	25 (46.3)
No (placebo)	29 (53.7)

OM, oral mucositis; RT, radiotherapy.

*All variables given as *N* (%), unless otherwise indicated.

The inclusion criteria for the trial were as follows: 1) histologically confirmed diagnosis of squamous cell carcinoma; 2) stage III–IV disease (7th edition, AJCC) (24); 3) tumor location in the oral cavity, oropharynx, larynx, hypopharynx, or nasopharynx; and 4) eligibility to receive chemotherapy (triweekly cisplatin or weekly cetuximab) plus radiotherapy. All patients should have level II lymph node involvement. Patients with salivary gland or sinonasal tumors were excluded.

Mucositis was graded according to the RTOG (21) and CTCAE v.4 scales (22). The RTOG scale was used to assess OM in the oral cavity because it better defines morphological alterations to the mucosa. The CTCAE was used to assess dysphagia (pharyngeal wall mucositis) because it provides a more accurate assessment of functional mucositis and odynophagia.

### Treatment characteristics

The treatment intent was either radical or postoperative (the latter only in patients with oral cavity tumors). The prescribed RT dose to the oropharynx and/or oral mucosa was required to be ≥66 Gy.

Cisplatin (100 mg/m²) was administered every 3 weeks, starting on day 1 of RT. Cetuximab was given as a loading dose of 400 mg/m² on day −7 (i.e., 7 days before the start of RT), followed by weekly doses of 250 mg/m² throughout the course of radiotherapy.

Radical intent RT was delivered with volumetric modulated arc therapy (VMAT). The study drug (Mucomel^®^) was administered orally five times per 24 h during treatment and thereafter until the onset of G1 mucositis.

### Simulation and volume design

All patients underwent contrast-enhanced simulation computed tomography (CT) with immobilization by a thermoplastic mask. The simulation CT slice thickness was 3 mm.

Definition of the target volumes was agreed upon by the primary investigator and researchers in accordance with the RTOG planning guidelines (25, 26).

**Figure 1:**
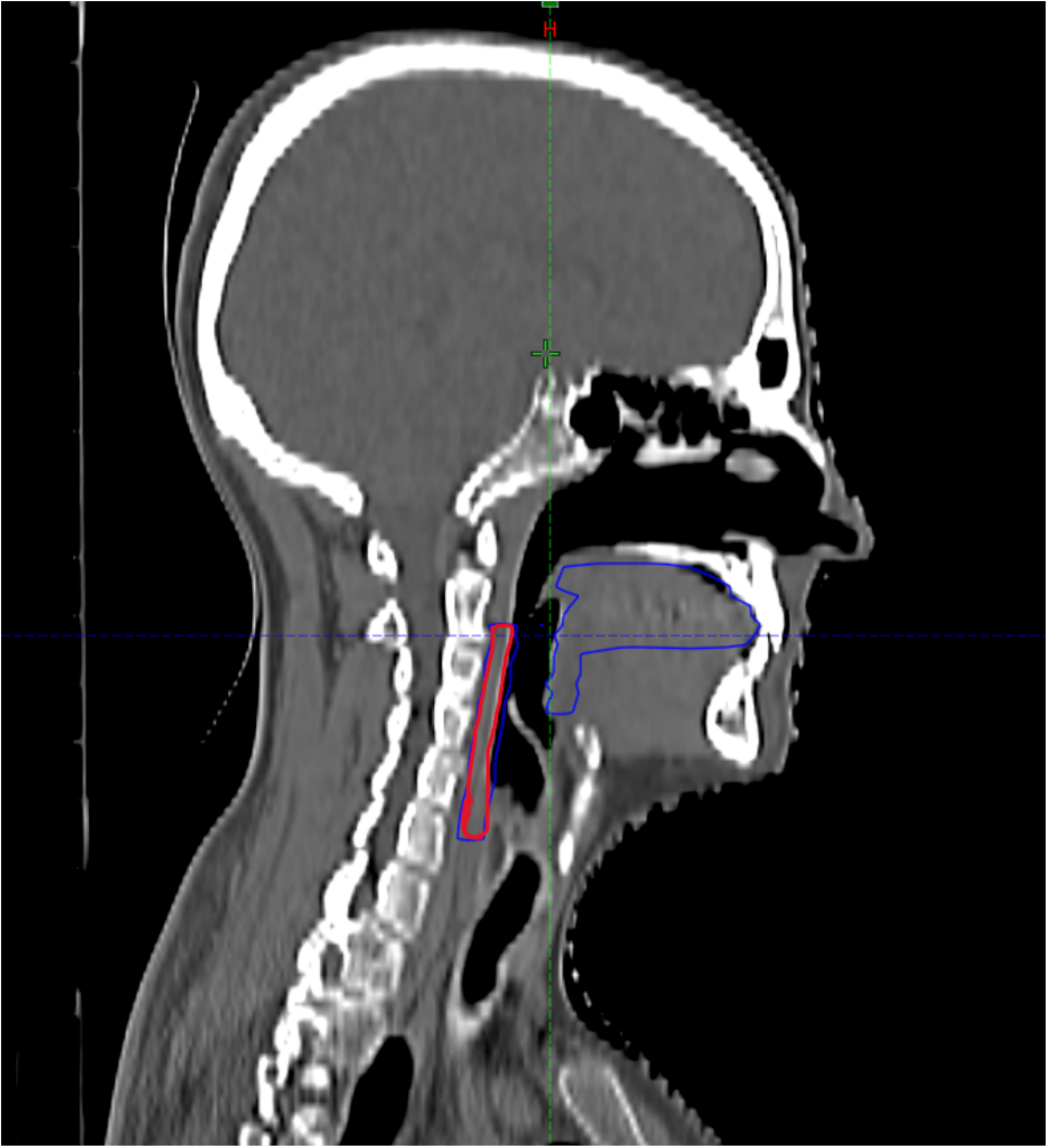
Contouring of the oral and pharyngeal mucosa in the TC plannigIn Red pharyngeal mucosa, in blue oral mucosa.

The oral cavity mucosa was divided into two separate organs at risk (OARs): the oral mucosa and the pharyngeal mucosa. The oral mucosa comprised the oral cavity and base of tongue, while the pharyngeal mucosa included the pharyngeal wall from the soft palate to the cricoid (**Figure 1**).

### Prescription and planning

Three dose levels of planning target volume (PTV1, PTV2, PTV3) were established based on the level of risk (high, intermediate, and low, respectively). In the high-risk regions (tumor and involved lymph nodes) (PTV3), the total prescribed dose was 69.96 Gy (2.12 Gy/fraction). The dose prescription for intermediate- and low-risk prophylactic regions was 59 Gy (1.8 Gy/fraction) (PTV2) and 54.12 Gy (1.64 Gy/fraction) (PTV1), respectively. In those patients who underwent surgery, the maximum dose to the surgical bed was 66 Gy (2 Gy/fraction). Dose normalization to the PTV was performed in accordance with the recommendation of the International Commission on Radiation Units (ICRU) number 83 (27). The consensus dose limits recommended by Brouwer et al. for OAR delineation were used (28).

### Outcome assessment

The two regions of interest (oral and pharyngeal mucosae) were photographed and assessed twice weekly (≥48 h between each assessment) to determine the mucositis grade. The assessments were performed separately by a medical oncologist and a radiation oncologist, both of whom were involved in patient follow-up during treatment.

We registered the mucositis grade (RTOG and CTCAE), date of onset, and date of change in grade or resolution of mucositis (or until week 12 from treatment initiation). All participants underwent training to assess mucositis in order to reduce the valuation deviation.

### Statistical analysis

We assessed the following clinical variables: age, sex, tumor location, stage, treatment duration, and type of systemic therapy (cisplatin or cetuximab). The dosimetric variables were extracted from the dose volume histograms (DVHs).

Dosimetric data for the oral and pharyngeal mucosae included the following: total dose; mean, median, and maximum dose; and accumulated dose at the onset of OM (G2 and G3). The percentage volume of each mucosa that received 5 to 70 Gy of radiation was measured in intervals of 5 Gy (V5 to V70). We assessed the total mucosal volume and the involved volume (volume included in the target area) for each mucosa for the three dose levels (PTV1: 54.12 Gy; PTV2: 60 Gy; PTV3: 66–69.96 Gy).

Fisher’s exact test or the Wilcoxon–Mann–Whitney test was used, as appropriate, to determine the association between G2/G3 OM and the clinical and dosimetric variables. The Shapiro–Wilk test was used to assess the distribution of the variables (normal or non-normal).

**Figure 2:**
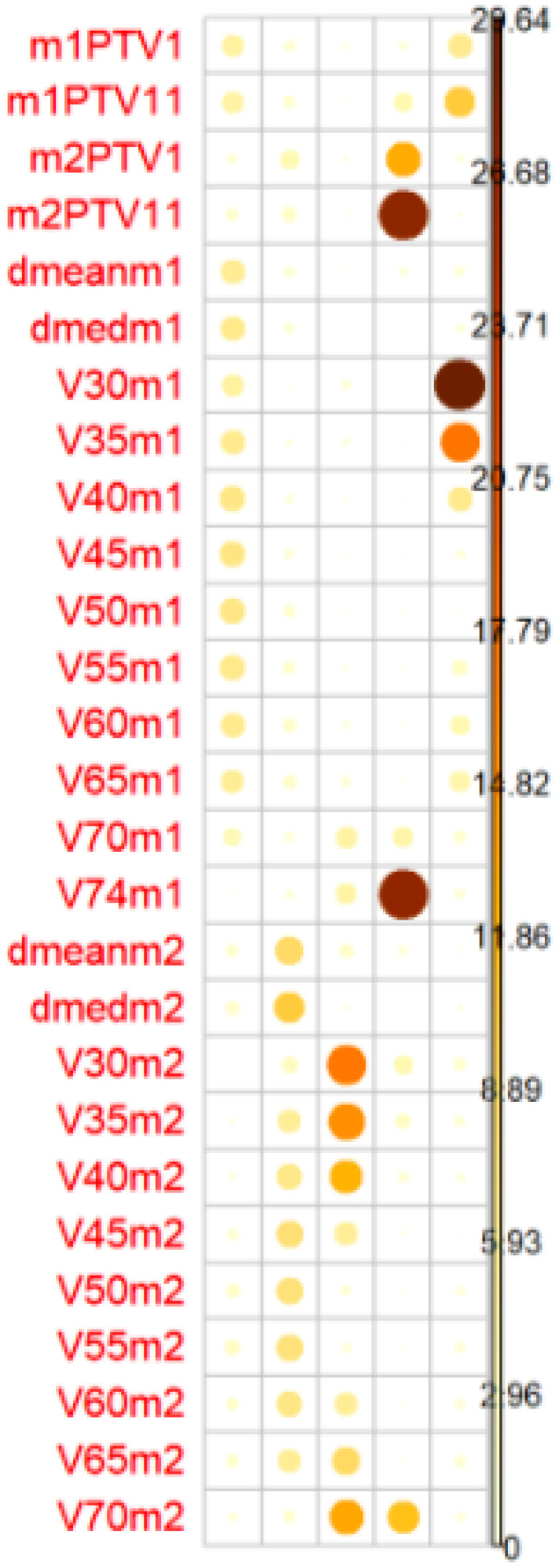
The balls colors determines the representation of the factors in the PC (principal components). PC1 represents dosimetrics factors of oral mucosa Mucosa 1 and PC2 dosimetrics factor of pharyngeal mucosa. Mucosa 2PTV1(54,12 Gy), PTV2 (PTV11 60 Gy), PTV3 (PTV111 66/69,96 Gy).

Univariate and multivariate regression analyses were performed. The multivariate regression included all categorical variables, non-correlated numerical variables, and correlated numerical variables using principal component analysis (PCA). For the PCA, we selected only the correlated numerical variables that were significantly associated with G2/G3 OM (*p* < 0.1 in the univariate regression analysis or Student’s *t*-test/Wilcoxon–Mann–Whitney test). The principal components needed to explain ≥85% of the variance were included (**Figure 2**).

The variables were introduced and removed one by one (stepwise) on the multivariate regression analysis to ensure that they improved McFadden’s pseudo-*R*-squared (*R*^2^). The final multivariate regression analysis included the following variables: oral treatment (Melatonin Gel^R^ or placebo), type of systemic therapy (cisplatin or cetuximab), tumor location, stage, sex, age, treatment duration, healthy volume for the oral and pharyngeal mucosae, PC1, PC2, PC3, and PC4.

## Results

### Patient characteristics

The clinical and demographic characteristics of the study population are presented in **Table 1**. The mean patient age was 59.7 years (range: 49–86). Among the 54 patients, 27 (50%) received cisplatin, and 27 (50%) received cetuximab. Twenty-five patients (46.3%) were treated with Mucomel®, while 29 (53.7%) received placebo.

The most common tumor location was the oropharynx (*n* = 28; 52%). Most patients (*n* = 49; 90.7%) had stage IVA–B disease (**Table 1**).

There were 52% of patients who developed mucositis in the oral cavity, measured by the RTOG scale (**Table 2**), and 44% in the pharyngeal mucosa, measured by the CTCAE.

**Table 2:** Incidence of mucositis G2–3 in both mucosae measured by the RTOG scale.

Grade	Oral mucosa (*n*/%)	Pharyngeal mucosa (*n*/%)
G3	28 (52%)	24 (44%)
G2	19 (35%)	26 (48%)

Dosimetric characteristics of the variables studied are presented in **Table 3**.

**Table 3:** Dosimetric values in the oral and pharyngeal mucosae.

Variable	Oral mucosa*	Pharyngeal mucosa*
Mean volume, cm^3^ (range)	77 (21.3–162.3)	22.2 (10–63)
% healthy mucosa included in PTV1	52% (37%–86%)	41% (0%–59%)
Mean RT dose	51 (24–69.96)	60 (36–69.96)
Median RT dose	50 (21.6–69.96)	59 (39–69.96)
Mean RT dose in patients with G3 OM	56.6 (29–69.96)	61.50 (45–69.96)
Median RT dose in patients with G3 OM	55 (21.2–69.96)	61 (50–69.96)
V35%	70 (21–100)	96 (53–100)
V50%	53 (34–100)	80 (21–100)
V65%	31 (0–99)	39 (0–97)
Healthy volume mucosa	52 (0–100)	41 (0–93)

OM, oral mucositis; G, grade; RT, radiotherapy.

*RT dose is given in Gy with dose range in parentheses.

*Healthy volume mucosa outside PTV1.

*V35% mucosa volume that received 35 Gy.

The administration of Melatonin Gel^R^ did not significantly reduce the incidence of G3 OM in the oral mucosa [*p* = 0.78; odds ratio (OR): 1.32; 95% confidence interval (95% CI): 0.4–4.45) or the pharyngeal mucosa (*p* = 0.32; OR: 4.06; 95% CI: 0.36–2.212) in this sample.

### Univariate analyses

#### Clinical variables

Cetuximab treatment was significantly associated with G3 OM in the oral mucosa (*p* = 0.013: OR: 0.21; 95% CI: 0.566–0.75). No association was observed between systemic therapy and G3 OM in the pharyngeal mucosa. Moreover, no significant association was observed between the presence of G3 OM in the oral or pharyngeal mucosae and any of the following variables: age, sex, treatment duration, tumor location, and tumor stage.

#### Dosimetric variables

**Figure 3:**
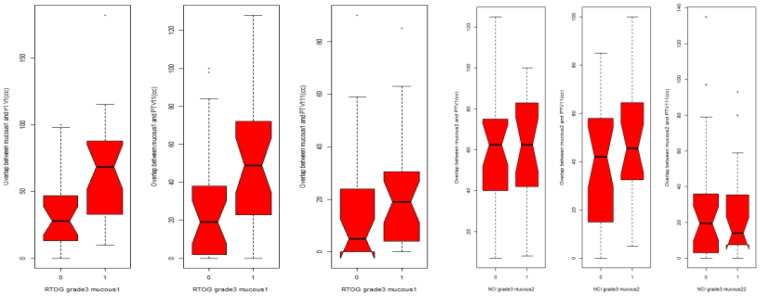
Box plot relationship between PTV1, PTV2(PTV11) and PTV3(PTV111) expressed as cm3(CC)with MG3 in oral mucosa and pharyngeal mucosa.0: patients without MOG3, 1: patients with MGO3RTOG for oral mucosa, CTCAE for pharyngeal mucosa.

The volume of the oral mucosa included in PTV1 (50 cc; *p* = 0.002) and PTV2 (40 cc; *p* = 0.012) was significantly associated with G3 OM (**Figure 3**). However, there was no association between G3 OM and the high-dose volume (PTV3; *p* = 0.07), nor for pharyngeal mucosa in any of the volumes (PTV1: *p* = 0.31; PTV2: *p* = 0.1).

All doses from V35 to V70 were significantly associated with G3 OM (*p* = 0.047). In the univariate analysis, the median dose (but not the mean or maximum dose) to the oral mucosa was significantly (*p* = 0.044) associated with G3 OM (**Table 3**). The volume of PTV1 (*p* = 0.003; OR: 1.03; 95% CI: 1–1.05), PTV2 (*p* = 0.022; OR: 1.02; 95% CI: 1–1.04), and V35 >70% (*p* = 0.048; OR: 1.02; 95% CI: 1–1.05) was significantly associated with G3 OM in the oral mucosa. All values from V35 to V70 were also significantly associated with G3 OM in the oral mucosa (**Table 4**).

**Table 4:** Univariate regression analysis showing the variables significantly associated with grade 3 OM in the oral mucosa.

Variable	*P*-value	95% CI	Odds ratio
CDDP	0.013	0.056–0.75	0.21
Median dose	0.044	1–1.1	1.05
PTV1	0.003	1–1.05	1.03
PTV2	0.022	1–1.04	1.02
V35	0.047	1–1.05	1.02
V40	0.033	1–1.05	1.02
V45	0.022	1–1.05	1.02
V50	0.022	1–1.05	1.02
V55	0.031	1–1.04	1.02
V60	0.044	1–1.04	1.02
V65	0.06	1–1.04	1.02
V70	0.031	1.01–1.12	1.06
Healthy tissue volume	0.023	0.95–0.99	0.98

OM, oral mucositis; CI, confidence interval.

CDDP as a protective factor against cetuximab.

The volume of healthy mucosa included in the PTV was significantly associated with G3 OM (*p* = 0.02; OR: 0.98; 95% CI: 0.95–0.99 (**Table 4**). The mean volume was 60 cm^3^ [standard deviation (SD): 30; range: 53–100]. By contrast, the volume of healthy mucosa in the pharyngeal mucosa was not associated with G3 OM in the pharyngeal mucosa.

### Multivariate analysis

**Figure 4:**
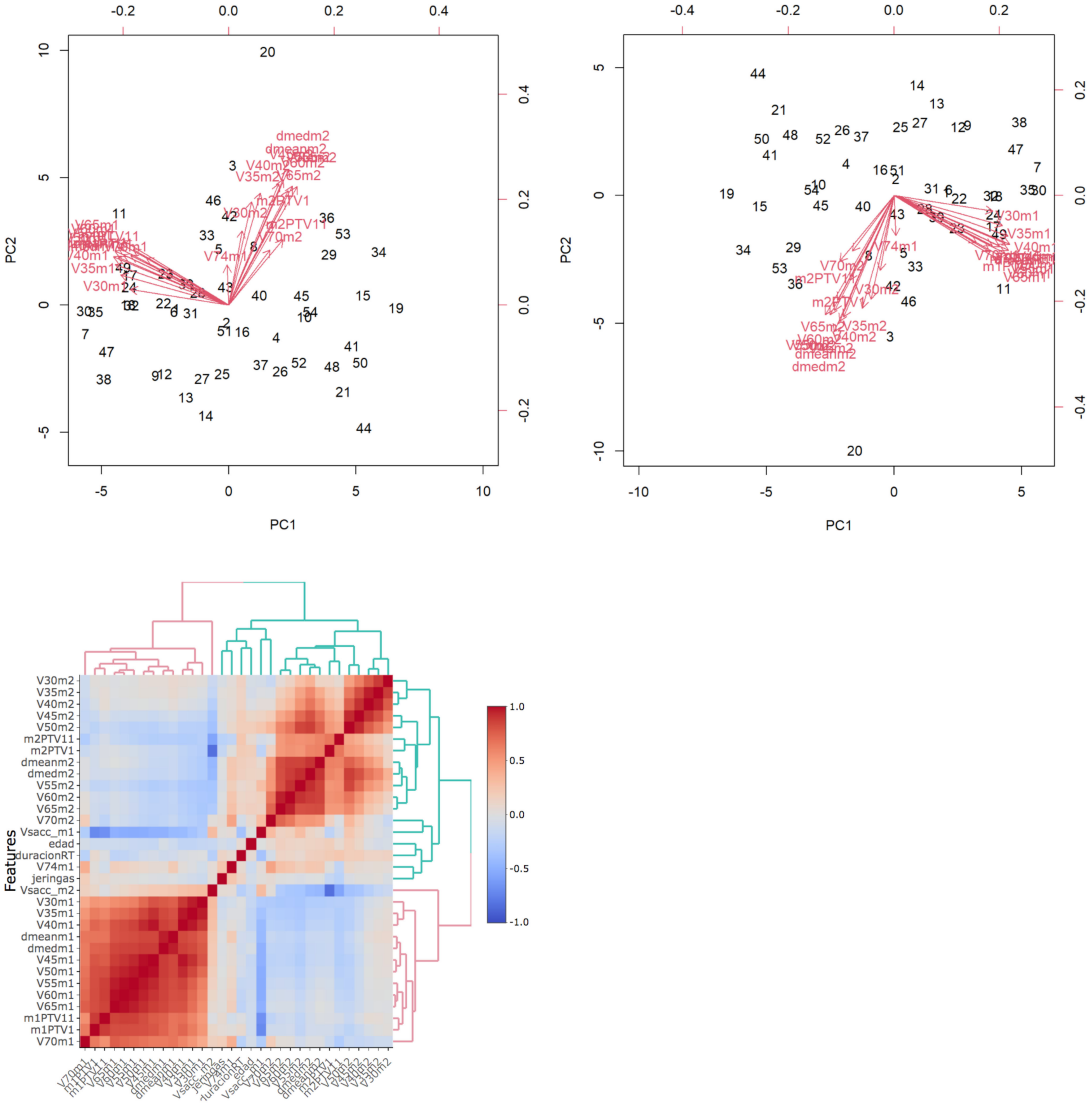
Biplot: graphical representation of dates as vectors of both mucosa and identification of relationship patterns. The common direction of the vectors represents the positive correlation between the variables.The parameters of each mucosa are highly correlated with each other. PCA is performed to correctly asses the incidence of highly correlated variables in mucositis. Heatmaps represents the relationship between clinical and dosimetric factors from both mucosae. Red represents high correlation while blue is low correlation. Oral mucosa parameters show high correlation, pharyngeal mucosa parameters have low correlation between them.

On the multivariate analysis, the following variables were significant risk factors for G3 OM in the oral mucosa: cetuximab treatment (*p* = 0.01), oropharyngeal location (*p* = 0.03), and PC2 (pharyngeal wall dosimetric variables) (*p* = 0.04) (**Figure 4**), with McFadden’s pseudo-*R*^2^ of 0.46 (G3 OM) and 0.44 (G2 OM). The absolute healthy volume of the oral mucosa was a significant protective factor (*p* = 0.03; McFadden’s pseudo-*R*^2^ = 0.46) (**Table 5**).

**Table 5:** Multivariate regression analysis of the oral mucosa.

Parameter	*P*-value	Odds ratio	95% CI
Cisplatin	0.0150	0.006	0.00004–0.181*
Hypopharynx	0.4029	7.236	0.0783–1,489.3
Larynx	0.1688	72.36	0.3557–7,747.03
Nasopharynx	0.0881	164.5	0.6451–15,533
Oropharynx	0.0257	135.3	3.7441–26,656*
Stage	0.4448	0.155	0.0008–16.956
Sex	0.0738	29.94	1.1393–2,557.5
Age	0.1655	0.937	0.8479–1.0247
Volume of healthy oral mucosa	0.0347	0.939	0.8772–0.9890*
Volume of healthy pharyngeal mucosa	0.6783	1.066	0.7860–1.4872
PC1	0.9905	1.003	0.5753–1.7834
PC2	0.0377	1.788	1.0826–3.3960*
PC3	0.4498	1.345	0.6308–3.1214
PC4	0.1617	0.327	0.0554–1.3484

* Statistically significant parameters.

The heatmap represents the relationship between clinical and dosimetric factors from both mucosae. Red represents high correlation, while blue represents low correlation. Oral mucosa parameters show a high correlation, while pharyngeal mucosa parameters show a low correlation.

None of the previous variables was associated with G2 OM. The model fit was considered good (McFadden’s pseudo-*R*^2^ = 0.44).

For the pharyngeal mucosa (McFadden’s pseudo-*R*^2^ = 0.28), the healthy volume of oral mucosa (*p* = 0.03) was a protective factor. Tumors located in the oropharynx were associated with a higher risk of G3 OM, which was close to reaching statistical significance (*p* = 0.06). None of the factors was associated with G2 OM in the pharyngeal mucosa.

In patients with G3 OM in the oral mucosa, no significant differences were observed between those treated with cetuximab or cisplatin in terms of the median radiation dose (40.45 vs. 38 Gy, respectively; *p* = 0.66; 95% CI: −9.43 to 14).

Dosimetric variables in oral mucositis G3 between patients with and without oral mucositis G3 were statistically significantly measured by the Wilcoxon rank test (**Table 6**).

**Table 6:** Dosimetric variables in patients with and without G3 mucositis in the oral mucosa.

Parameters	Mucosa with mucositis G3	Mucosa without MG3	*P*-value
Median dose	55 (32–70)	44 (29–70)	0.04
PTV1	68.5 (10–180)	28 (0–100)	0.001
PTV2	49 (0–128)	19 (0–100)	0.011
V35	85.5 (32–100)	66.5 (25–100)	0.06
V40	75 (20–100)	55.5 (16–100)	0.04
V45	66.5 (12–100)	48 (11–100)	0.02
V50	60.5 (7–100)	35 (4–100	0.01
V55	50.5 (3–100)	26 (0–100)	0.009
V60	41 (0–100)	18.5 (0–100)	0.015
V65	35 (0–99)	14.50 (0–95)	0.027
V70	9.5 (0–49)	2.5 (0–28)	0.017
Volume of healthy mucosa	20 (0–120)	46.5 (0–163)	0.001

- Cisplatin is a protective factor against cetuximab.
- Oropharyngeal location is a risk factor.
- PC2, which represents dosimetric factors for pharyngeal mucosa, is a risk factor for MOG3 in M1.
- A large volume of healthy mucosa outside the PTV1 may be a protective factor for mucositis.

## Discussion

The introduction of IMRT as a standard technique in the treatment of head and neck cancer allows for a greater confirmation of the high-dose region, with two important implications for toxicity: first, it allows for lower doses to OARs; second, it tends to expand the volume of OARs exposed to lower levels of radiation. In this context, it is important to find a reliable predictor of mucosa acute toxicity.

In our study, severe (≥G3) OM was significantly associated with several factors: oropharyngeal tumor localization, cetuximab treatment, oral mucosa receiving ≥35 Gy (V35 > 70%), and the volume of the healthy mucosa exposed to low and intermediate radiation doses (PTV1: 50 cm^3^, PTV2: 48 cm^3^). Notably, none of the dosimetric plan parameters were associated with G3 OM in the pharyngeal mucosa. To our knowledge, this is the first study to specifically evaluate the risk of developing severe OM in the oral and pharyngeal mucosae following concomitant RT and systemic therapy within the framework of a clinical trial.

The incidence of severe mucositis in our cohort was 52% for the oral mucosa and 44% for the pharyngeal wall. These figures are consistent with previously reported rates in oral mucositis (43%–56%) (1, 2, 9, 15). However, published incidence rates vary widely in the literature, ranging from 20% to 78% (17, 29).

In the case of pharyngeal wall mucositis, the incidence of G3 observed in our study (44%) was lower than the incidence reported by Bhide et al. (16) (61%–87%) in their retrospective study based on the data from two dose escalation trials. By contrast, Mazzola et al. reported a substantially lower incidence rate (18%) (18).

This large variability in reported incidence rates may be due to several aspects. One of them is the use of different scales for the assessment of mucositis.

Although the WHO scale (30) is the most widely used for assessing mucositis, we selected the NCI CTCAE (22) and RTOG scales (21), as they better matched the specific aims of our study. Villa et al. (31) showed strong correlations among these three most common scales when assessing severe OM (≥G3). In their study, 99.6% and 97.7% of patients with severe OM according to the WHO scale were also classified as G3–G4 on the CTCAE (*κ* = 0.98) and RTOG (*κ* = 0.69) scales, respectively. However, concordance was lower at the extremes of severity.

Comparing these scales is further complicated by ongoing updates, especially to the CTCAE, and by variability in evaluator training and experience. To reduce grading inconsistency, we assessed the oral and pharyngeal mucosa separately, using RTOG for the oral cavity and CTCAE for the pharyngeal wall. All evaluators underwent dedicated training as part of the clinical trial protocol.

We found no association between severe OM and the patient-related factors such as age, sex, treatment duration, Mucomel^R^ intervention, or tumor stage. In the multivariate analysis, oropharyngeal tumors were significantly associated with a higher incidence of G3 OM in the oral mucosa. This may be explained by the fact that oropharyngeal tumors were the most frequent in our cohort (52%) and tended to have the largest tumor volume, and most of these cases (91%) were locally advanced disease (stage IVA–B).

Previous studies focusing exclusively on oropharyngeal tumors have reported highly variable rates of severe OM, ranging from 78% to 100% (17, 32), often using heterogeneous fractionation schedules (17, 32, 33). In contrast, our clinical trial applied standardized fractionation protocols aligned with current standards: moderately hypofractionated RT (2.12 Gy) in non-surgical patients and conventional fractionation (2 Gy) in those treated surgically.

**Figure 5:**
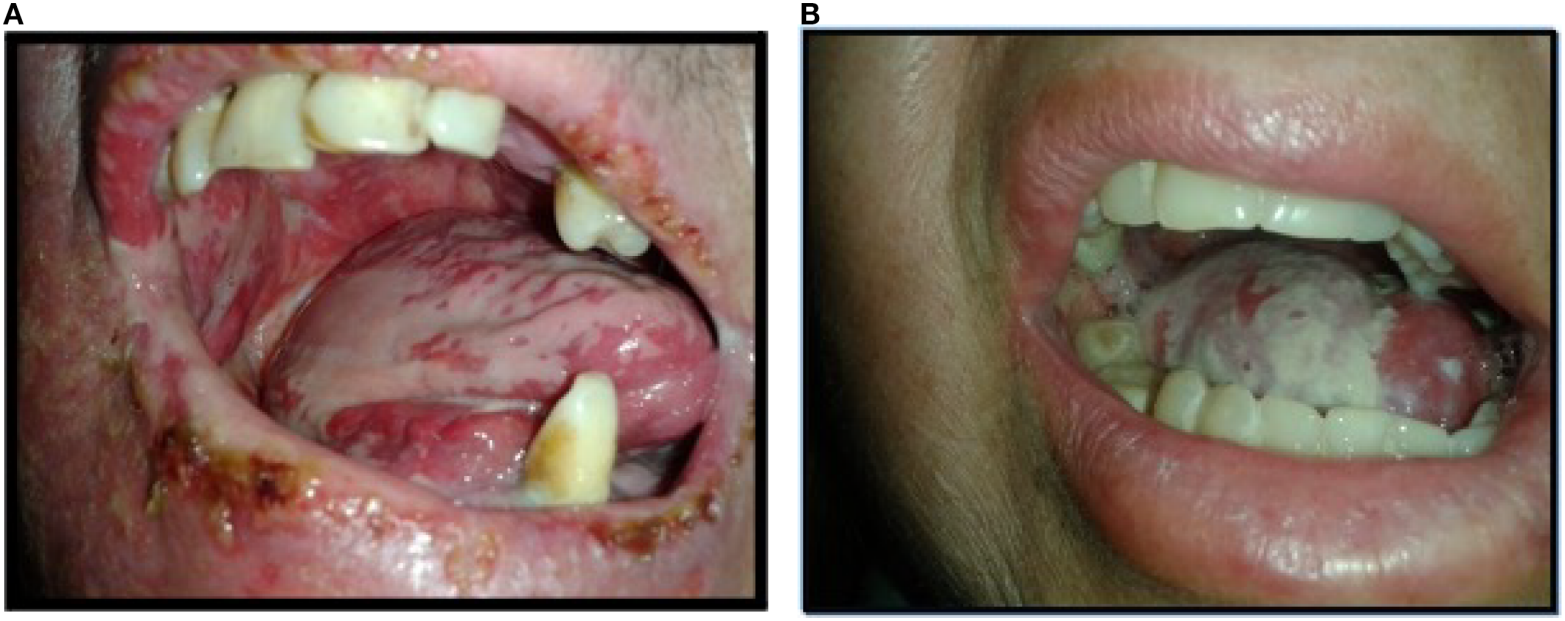
Mucositis G3. Image **(A)** Oral mucositis G3 in patient treated with IMRT plus cetuximab. Image **(B)** Oral mucositis G3in patient treated with IMRT plus CDDP.

The incidence of grade 3 OM was significantly higher in patients treated with cetuximab compared to those receiving cisplatin (70% vs. 33.3%, *p* = 0.013) (**Figure 5**), with OM being 2.5 times more frequent in the cetuximab group. These results contrast with most previous studies, which reported no significant differences between the two treatments. For instance, Bonner et al. (34) observed similar rates of G3 OM with RT alone and RT plus cetuximab (52% vs. 56%; *p* = 0.44), while Gillison et al. (35) and Lefebvre et al. (36) found nearly identical rates between cisplatin and cetuximab (41% vs. 46% and 43% vs. 43%, respectively). Other studies have reported even lower rates of severe OM with cetuximab, such as 13% in one case, although Ang et al. (37) showed that adding cetuximab to RT and cisplatin increased the rate of G3–4 OM (43.2% vs. 33.3%) without improving survival outcomes. Notably, our findings are in line with those of Gebre-Medhin et al. (38), who, in a phase III trial, also reported a significantly higher incidence of G3 OM in patients receiving weekly cetuximab compared to cisplatin (*p* = 0.035). The underlying mechanisms for this increased toxicity are not fully understood but may involve a reduction in epidermal growth factor (EGF) during RT, as suggested by previous studies (4, 5, 41) along with greater exposure of radiosensitive healthy mucosa to low and intermediate doses due to IMRT.

Some aspects that could explain the different results obtained in our study compared to those published, such as the general health status of the patients and the tumor stage, were not different in both groups. To be included in the main trial, patients had to present a performance status (PS) of 0–1. However, the impact of comorbidities on the development of mucositis has not been evaluated. In terms of tumor stage, 100% of patients treated with cetuximab had stage IV disease compared to 90% of patients treated with cisplatin.

One of the factors that could have influenced the different results is that, in this study, the main objective was oral mucositis, and its evaluation was more specific using two different scales.

In terms of dosimetric factors according to the plan, ≥G3 OM in the oral mucosa was associated with the median dose (56 Gy) and the volume of the oral mucosa receiving low and intermediate doses. Other authors reported a similar association between the mean dose (51 Gy) and G3 OM (18, 32). By contrast, in our study, a mean dose of 54 Gy (range: 24–69.96) was not significantly associated with severe OM (OR: 1; 95% CI: 0.99–1.09).

In contrast to other studies, we did not observe an association between the maximum dose and ≥G3 OM reported by other authors in oral mucosa (39) and intestinal mucosa (40). An explanation for these results could be that our patients received a homogeneous maximum dose.

We reported a strong correlation between dose volume parameters and the development of G3 OM in the oral mucosa, particularly when V35 exceeded 70%, albeit with slightly different thresholds, for example, V45 >40% (18) and V30 >72% (29). Taken together, with the statistical significance of the volume within PTV1 and PTV2, these findings suggest that limiting low-dose radiation exposure to the oral mucosa may reduce the risk of G3 OM. The significant differences in dosimetric factors between patients with and without G3 OM in our cohort support this interpretation, as do the findings from Liu et al. (9) in patients with nasopharyngeal carcinoma. Additionally, a larger volume of healthy mucosa outside the PTV appears to act as a protective factor against the development of severe OM.

PC2 (pharyngeal wall dosimetric factors) was significantly associated with G3 OM (*p* = 0.037; OR: 1.79; 97.5% CI: 1.08–3.40), but not with pharyngeal mucositis. This association was likely attributable to the high volume treated and the predominance of oropharyngeal localization in our cohort.

Several factors have been associated with an increased incidence of ≥G3 mucositis of the pharyngeal wall, often reported as acute dysphagia in the literature, including doses ≥50 Gy (10, 42), pharyngeal wall length >8 cm (16), V50 >70% to the median constrictor (*p* = 0.05) (16), and the extent of mucosal surface receiving ≥1 Gy per fraction (15). In a retrospective study of 144 patients treated with various radiotherapy fractionation schemes, Bhide et al. (33) identified a dose threshold of 44.5 Gy for inducing G3 acute dysphagia in 50% of cases, with each additional Gy increasing the risk by 2.4%. The discrepancy between our findings and those reported in previous studies may be attributed to differences in dose distribution between the two mucosal sites. In our cohort, the pharyngeal wall received consistently higher doses than the oral mucosa, with V35 ≥96% vs. 70%, V50 ranging from 80% to 53%, and median doses of 59 Gy vs. 50 Gy, respectively. Additionally, the proportion of uninvolved mucosa was lower in the pharyngeal wall (41%) compared to the oral cavity (52%). This contrast could be partly explained by the high prevalence (91%) of locally advanced oropharyngeal cancer (stage IVA–B) in our cohort, which required including a significant volume of both mucosal subsites. The use of a specific scale for functional mucositis such as CTCAE v.4 may have contributed to this result.

In conclusion, our study reinforces that severe oral mucositis is primarily influenced by dosimetric parameters (9, 18, 29, 33, 39) and systemic treatments such as cetuximab, with no clear association observed for the pharyngeal mucosa. These findings highlight the importance of focusing on toxicity reduction strategies and reducing the volume of healthy mucosa treated on the oral cavity mucosa and underscore the need for prospective studies to validate these associations.

This study has several notable strengths. It is based on data from a randomized clinical trial specifically designed to evaluate the incidence of mucositis, ensuring high methodological rigor and standardized procedures for mucositis grading, volume delineation, and radiotherapy dosing. Unlike many previous studies, the treatment protocol was homogeneous across all patients. Importantly, the role of cetuximab in the development of mucositis was a primary outcome, whereas earlier studies primarily focused on survival. This is also the first study, to our knowledge, to directly compare the impact of cetuximab versus cisplatin on oral mucositis. Another key strength is the separate evaluation of oral and pharyngeal mucosa, which allowed for a more nuanced analysis of clinical and dosimetric influences. The main limitation is the relatively small sample size (*n* = 54), which may reduce the power to detect subgroup differences based on treatment type or tumor location. Additionally, while the inclusion of Melatonin Gel^R^ as a potential protective agent could have influenced the results, prior analysis demonstrated no significant impact on mucositis incidence.

Further studies with larger patient cohorts will be needed to validate our findings.

## Data Availability

The raw data supporting the conclusions of this article will be made available by the authors, without undue reservation.
